# Introduction of a formative assessment tool in a post-graduate training program in India: a mixed methods evaluation

**DOI:** 10.1186/s12245-024-00604-6

**Published:** 2024-03-01

**Authors:** Katherine Douglass, Tania Ahluwalia, Brianna McKiernan, Heena Patel, Natasha Powell, Jacob Keller, Serkan Toy

**Affiliations:** 1 Medical Faculty Associates, Department of Emergency Medicine, The George Washington University, 2120 L Street NW, Ste 450, Washington, DC 20037 USA; 2 Children’s National Hospital, Washington, DC USA; 3 George Washington University School of Medicine & Health Sciences, Washington, DC USA; 4 Virginia Tech Carilion School of Medicine, Roanoke, VA USA

**Keywords:** Medical education & training, Accident and emergency medicine, India

## Abstract

**Background:**

Our institution has longstanding post-graduate education and training partnership programs in Emergency Medicine (EM) across India. A programmatic challenge has been the integration and uptake of evidence-based medicine and lifelong learning concepts. Formative assessment (FA) is intended to enable learners to monitor learning, identify strengths and weaknesses, and target areas of growth. As part of a program improvement initiative, we introduced an online FA tool to existing summative assessments. This study investigates how the FA tool was used and perceived by trainees.

**Methods:**

246 trainees across 19 sites were given access to the FA tool. Usage metrics were monitored over 12 months. Semi-structured interviews were conducted in person with trainees using a purposive sampling methodology. A hybrid thematic analysis approach was used to determine themes. Interviews were coded independently by two blinded researchers using NVivo software. The study was deemed exempt by our institutional review board.

**Results:**

There was high variability in trainees’ utilization of the FA tool. Trainees who used the FA tool more performed better on summative exams (*r* = 0.35, *p* < 0.001). Qualitative analysis revealed that trainees were motivated to learn for improved clinical knowledge and to be a good physician, not only passing exams. Benefits of the tool included the relationship to clinical practice and thorough explanation of answers, while disadvantages included topics unrelated to India.

**Conclusion:**

The integration of a FA tool has provided positive outcomes for trainees in EM education programs in India. Lessons learned may apply globally to other contexts and programs.

**Supplementary Information:**

The online version contains supplementary material available at 10.1186/s12245-024-00604-6.

## Background

An aspirational goal in effective medical education is to foster the practice of self-motivated learning amongst students. Self-regulated learning is defined as learners’ active participation in their own learning process from metacognitive, motivational, and behavioral perspectives [1]. This is particularly important as physician trainees go on to independent clinical practice where updated information is constantly available that must be located, synthesized and applied to a clinical scenario.

Formative assessment (FA) is intended to monitor and encourage student learning, helping students to identify their own academic strengths and weaknesses. FA is often described as low stakes and designed to inform students and teachers, a change from the more traditional summative assessment, which is a high stakes evaluation process designed to measure students against a benchmark. Educational programs are evolving in their utilization of assessment methodologies, moving from assessment of learning to assessment for learning [2]. Integration of formative assessment can be considered necessary in higher education for effective student learning, specifically related to the conceptual framework of self-regulated learning [3–6]. Furthermore, it is important to consider cultural context and potential differences as an essential piece of this puzzle [7, 8]. 

In 2006, our institution started our first partnership education and training program in Emergency Medicine together with a local institution in India. Since that time, our programs have changed and evolved substantially, as we have increased in number and location, but also made changes to the delivery of education and training [9]. At the time of this study, there were 246 trainees enrolled at 19 sites, in a longitudinal three year residency like program. Initially, traditional summative assessments (written, multiple choice questions) were used on a monthly and annual basis to evaluate students’ performance, in addition to a high stakes exit exam at the end of the program (including written and practical exam components). Various factors initially pushed our programs towards even more frequent summative exams, given the desire to validate the teaching and learning effectiveness in an otherwise non-traditional international partnership program model. However, informal and formal program evaluations revealed the dissatisfaction of all stakeholders with this model, including trainees, in-country faculty, and international faculty. From the students’ perspective, this was in part due to the expressed desire for more explanation regarding questions, correct and incorrect answers, and the opportunity to learn more from the exam experience. In 2018, in response to trainee and faculty member feedback regarding existing assessment methods, we introduced an online FA tool as an integrated component of the curriculum in addition to and replacing some of the ongoing summative assessment.

Prior study regarding the integration of formative assessment in India has been limited. The traditional climate and culture of medical education in India relies on hierarchical teaching and practice, typically more reliant on summative examination strategies. Students are often taught to learn for the exam, and that the most senior person in the room is correct [10]. This climate is not unique to India and can pose significant challenges with teaching concepts such as life-long learning and evidence-based inquiry. One qualitative study showed variable opinions among post-graduates and faculty members regarding the integration of FA [11]. Integration of real time feedback is often an essential component of FA, one that is often still new in medical education settings in India [12]. 

Rosh Review is a commonly used study resource for Emergency Medicine trainees [13]. While often used as an exam preparation tool, the breadth of questions and associated resources allow for and promote flexible and adaptive use. The question bank allows users to access questions in specific topic areas, and users can choose either tutor mode or exam mode to curate adaptable experiences fine-tuned for individual learning needs. Questions are designed to not only grade an answer as right or wrong, but provide explanation regarding the correct and incorrect answers, with additional opportunity to deepen learning through expanded resources and references. The choice of this resource was in response to program feedback regarding desire for explanations and deeper learning linked to exams.

This study was undertaken to understand faculty and trainee perceptions regarding the newly introduced FA tool, partially in relationship to the utilization of the tool and performance. Specifically, we sought to obtain data from the usage of a newly introduced FA tool to determine the relationship between usage and performance on summative assessments. We also sought to understand different perspectives and rationales for frequent or infrequent usage of the tool among different users. While the goals of this study are specific to the program described, the bigger picture goal is to find applicable lessons regarding integration of FA that could be applied beyond this specific program.

## Methods

An existing FA tool (Rosh review) was identified and aligned with an existing 36-month modular curriculum [13]. 246 trainees across 19 sites were given access to the FA tool with both planned quizzes and additional available questions. Monthly quizzes were developed by program faculty, aligned with the 36-month modular curriculum, with expected completion as a program requirement. Trainees were provided with an introduction to the FA tool including both quizzes and the full available question bank. Additional question bank access and usage was optional. Anonymous study numbers were assigned to each individual. Usage metrics were monitored over the initial 12 months. Based on basic usage metrics, three separate groups were identified: high performers (> 80% of questions answered correct), high utilizers (top 15% of number of questions answered) and low utilizers (bottom 15% of questions answered). To confirm this initial list, Ward’s Method, a multivariate hierarchical cluster analysis, was used to explore whether the trainees would form homogenous clusters based on their utilization (number of questions viewed (defined as question impressions) and total questions answered) of this educational resource [14, 15]. This list, then, was used to guide the purposive sampling.

A set of analysis of variance (ANOVA) tests were used to examine differences in the utilization of the educational tool between various subgroups. A bivariate Pearson correlation was used to examine the relationship between the utilization of the question bank and performance on regularly scheduled summative assessments. All statistical analyses were conducted with Statistical Package for the Social Sciences (IBM SPSS Statistics for Mac, Version 25.0; IBMCorp, Armonk,NY), with significance level set at *P* < 0.05.

The interview guide was developed by the research team based on prior literature as well as prior experience and informal conversations with users, following the interview protocol refinement framework [16]. Two medical student members of the study team who were trained as part of study preparation but had no previous experience or exposure to the programs or the individuals conducted the interviews. These two medical students traveled to India to conduct the interviews in person, at specific sites identified by willingness to participate combined with convenience.

The interview guide was piloted, revised, then finalized (see supplementary file). Interviews were conducted in person with trainees using a purposive sampling methodology to include individuals who were using the tool in different patterns. Interview participants were approached in person by one of the medical students to participate at a mutually agreed upon time. Verbal consent was obtained from interview participants. The interviews were conducted in English in a private environment without interruption. Interviews generally lasted 15–20 min. Interviews were recorded and transcribed without inclusion of individually identifiable information. Audio recordings were deleted upon completion of transcriptions.

A hybrid thematic analysis approach was used to guide a staged process for coding the interview data and identifying the themes [17]. Interviews were coded independently by two members of the research team using NVivo qualitative research software. The coding scheme can be found in Table 1.

**Table 1:** Coding Scheme

Theme	Sub-theme
Benefits of Rosh Review	Relationship to clinical practice; Learning style; Style of information; Explanation of answer choices; Level of information; Accessibility
Learning Strategies and Metacognition	Books; Practice questions; Instructors; Patients; Peer discussion; Websites; Apps
Use of Rosh Review	Learning tool; Number of questions; Assessment; Time; System based vs. mixed questions
Outside Resources	Study groups; Textbooks; Websites
Motivation	Passing exams; Clinical knowledge; Being a good physician; Self-regulated learning
Success	Passing exams; Learning; Application to clinical practice; Percentage or number of correct questions
Disadvantages of Rosh Review	Relevance in India; Challenges; Clinical relevance in India; Subjects specific to India not covered enough
Cultural Context	Education culture; Hierarchy

The design and reporting of data were based on the consolidated criteria for reporting qualitative research (COREQ) guidelines [18]. The study was deemed exempt from review by the institutional review board at our institution, and as part of that assessment was deemed not require local IRB approval due the low risk, educational nature of the project.

## Results

The demographics of the users is shown in Table 2, showing representation across program sites and post-graduate years.

**Table 2:** Demographics – *N* = 246

Variable	N	Percent
Institution; City		
Aster Medcity; Kochi	20	8.1
Aster CMI; Bangalore	13	5.3
Aster DM; Wayanad	5	2
AMRI Hospital; Bhubaneswar	21	8.5
Baby Memorial Hospital; Calicut	13	5.3
Believers Church Medical College Hospital; Thiruvalla	10	4.1
KDAH Hospital; Mumbai	26	10.6
Medica SuperSpecialty Hospital; Kolkata	4	1.6
Meenakshi Mission Hospital; Madurai	29	11.8
Moolchand Medcity; New Delhi	17	6.9
Peerless Hospital; Kolkata	30	12.2
Malabar Institute of Medical Sciences; Kottakkal	4	1.6
MAX Hospital; Dehradun	11	4.5
MAX Hospital; Mohali	1	0.4
MAX Hospital; Patparganj, New Delhi	10	4.1
MAX Hospital; Saket, New Delhi	10	4.1
MAX Hospital; Shalimar Bagh, New Delhi	8	3.3
MAX Hospital; Vaishili	4	1.6
Malabar Insitute of Medical Sciences; Calicut	10	4.1
Post-graduate year		
1	54	22
2	97	39.4
3	95	38.6

There was high variability among trainees in the extent of utilization of the question database. A one-way ANOVA showed a significant effect for training level, F(2,243) = 20.06, *p* < 0.001, and a post-hoc Tukey test indicated that PGY3s utilized this educational platform significantly more frequently compared to PGY1s & 2s as indicated by a higher number of total questions answered (*p* < 0.001)(see Table 3).

**Table 3:** Overall Means (SDs) – PGY comparisons

Variable	Training	N	Mean	SD	95% CI for Mean			Min		Max	
Total Question Impressions*	PGY 1	54	695.83	852.90	463.04	928.63	173		5721		
	PGY 2	97	747.77	540.07	638.93	856.62	76		2560		
	PGY 3	95	1651.4	1566.95	1332.2	1970.6	235		8608		
	Total	246	1085.33	1190.17	935.87	1234.8	76		8608		
Total Questions Answered	PGY 1	54	399.81	306.33	316.2	483.43	96		1597		
	PGY 2	97	465.49	335.16	397.94	533.05	76		1874		
	PGY 3	95	964.35	931.35	774.62	1154.07	90		4254		
	Total	246	643.72	680.20	558.3	729.14	76		4254		

* Impressions = Number of questions viewed

Trainees who used the database more performed better on summative exams as shown by the positive significant correlation between number of questions answered and the final, end of year exam score (*r* = 0.35, *p* < 0.001).

**Fig. 1:**
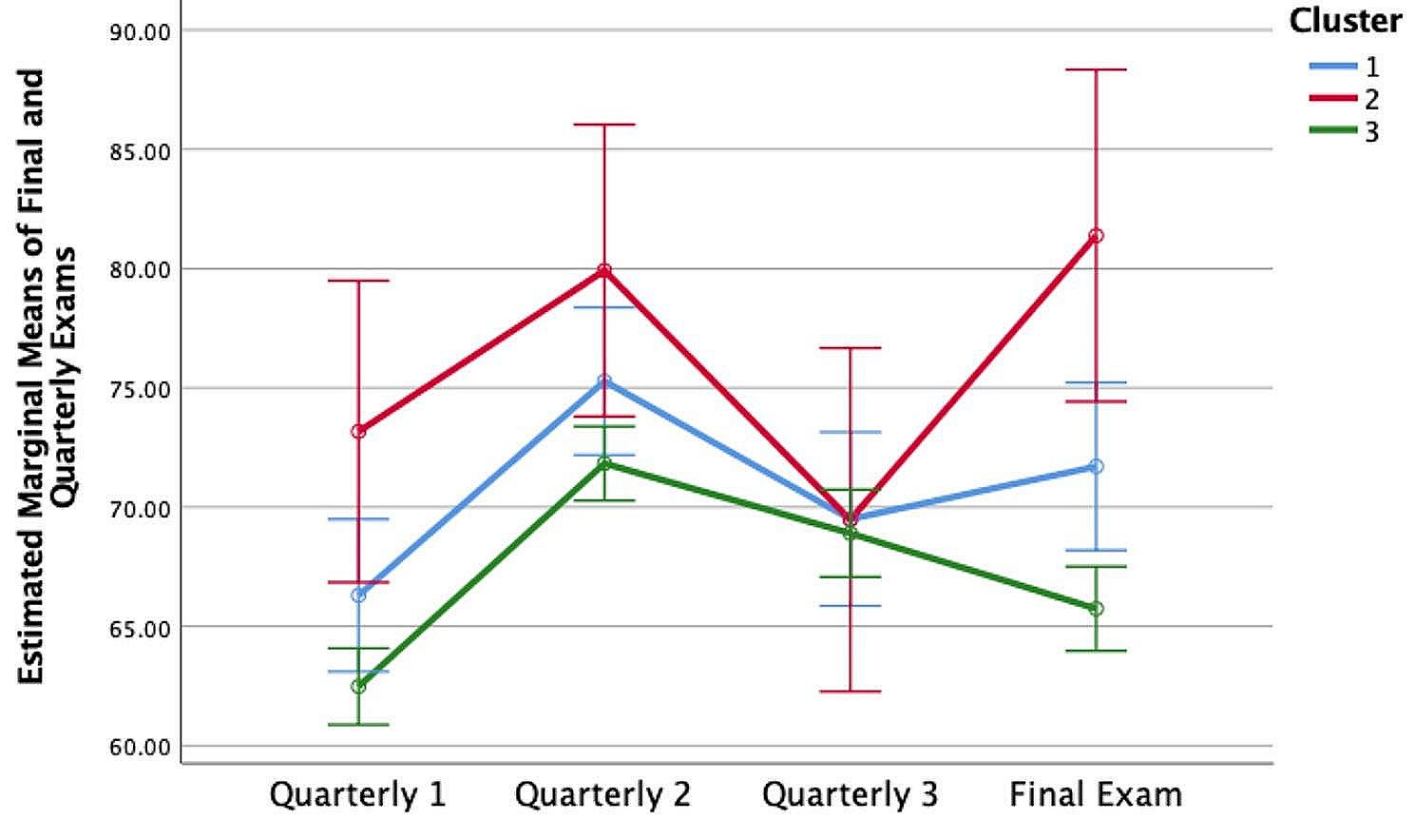
Comparison of clusters across one final and three quarterly exams. Error bars indicate 95% confidence intervals

Analysis of all trainees showed a natural division into three cluster groups that correlated well with our original group divisions. Cluster 1 (*N* = 47) consists of individuals who made average use of the question bank, while Cluster 2 (*N* = 12) is the group of high utilizers, and Cluster 3 (*N* = 187) is the group of low utilizers. The high utilizer group, Cluster 2, had significantly higher final exam scores than those of average utilizers, Cluster 1, (*p* = 0.046), and low utilizers, Cluster 3, (*p* < 0.001). Average utilizers, Cluster 1, outperformed low utilizers, Cluster 3, in the final exam (*p* = 0.009). Figure 1 depicts the differential performance of the three cluster groups on summative exams, showing alignment of usage with summative performance.

39 semi-structured interviews were conducted at six different sites. This included 13 high utilizers, 7 low utilizers, and 9 high performers. Ten interviews were conducted with individuals that did not fall into one of the predetermined groups. Analysis revealed four major themes among the interviews: motivation, benefits of the FA tool, disadvantages of the FA tool, and definitions of success. The majority of participants were motivated to learn for improved clinical knowledge (41%) and to be a good physician (24%), less were focused primarily on passing exams (16%). The benefits of the FA tool included the relationship to clinical practice (24%) and a thorough explanation of answer choices (22%), in addition to the level of information that is presented (15%). Disadvantages included unrelated topics to practicing in India (30%) and some topics being less covered than others (30%). Participants defined success as passing exams (39%) and application to clinical practice (32%). See Table 4 for theme related illustrative quotes.

**Table 4:** Illustrative quotes from interviews

Theme	Number of related comments (% of total comments within theme)	Representative comments
*Motivation*		
- Increased clinical knowledge	26 (41%)	“And for the studying point of view, I go about it case-based, like if I have seen a case of stroke today then I might go home and try to recall what did I do, what all should be done. “
- To be a good physician	15 (24%)	“Basically to learn and to read and it is not for clearing the exams it is to help myself get better in it and in clinical practice cause in a way it will help me in the long run.”
- Self-directed learning	12 (19%)	“When we go wrong we can go through the explanations so we learn and I think learning at the end of the day is more important than just the exam.”
*Benefits of the formative assessment tool*		
- Relationship to clinical practice	33 (24%)	The FA tool “has helped me in this transition from memorization-based study to practical application-based study.”
- Thorough explanation of answer choices	31 (22%)	“At the end of the day even if you are wrong, you learn something from it.”
- Style of information that is presented	22 (16%)	“So the main thing is explanation with picture, video and sounds. And also examinations including videos.”
*Disadvantages of the formative assessment tool*		
- Unrelated topics to practicing in India	20 (30%)	“Most of the tests that have been mentioned in the *FA tool* or in the text are usually not available or they take time. For example, the most common test that we usually see or read is D-dimer, but the results of D-dimer are usually available after three days for us and that does not remain for clinical significance…even if we send them it usually takes 3 or 4 days for that and patients cannot wait for that.”
- Some topics being less covered than others	20 (30%)	“More tropical diseases. More region specific. We have one subject called community medicine in our undergrad, so if *the FA tool* could have something like that. Because that actually brings up the entire community picture of our country, the major things that we face, so that will help.”
*Definitions of success*		
- Passing exams	16 (39%)	“Success for me is not percentage, it is for me that I have faced the questions myself.” “The score does matter in a way but not so much because at the end of it you still get an explanation and you still learn something new.”
- Application to clinical practice	13 (32%)	“If I have a sick patient which I wasn’t able to handle before if I was able to handle it after reading the *FA tool* because I remember something from there.”

## Discussion

This study provides an initial view of the introduction of a FA tool into an EM education program in India. While the statistical alignment of question completion and improved performance on exams is reinforcing for some, of more interest and relevance is the improved understanding of why and how the trainees chose to use the tool. The results of the qualitative data reveal that trainees are generally using the tool for learning and improved application to clinical practice, which is an exciting outcome for any education and training program, particularly in the global context. Increased usage of the tool also correlates with improved exam performance, which may reasonably serve as an additional incentive for increased uptake particularly for senior trainees studying for their exit exams. Past research describes the challenges associated with attributing success on summative exams to participation in additional quizzes, despite evidence of correlation [19]. Similarly, it is challenging to transition knowledge and practice of self-regulated learning from the theoretical to the clinical [20]. However, even initial trends towards students working and learning in this direction is positive.

The application of the educational concept of self-regulated learning in the cultural context of India has been particularly challenging over the years of programmatic development. Often the hierarchical nature of medicine in India lends itself more often to the notion that the oldest and most experienced person in the room is always right. This top-down approach permeates the education and training environment and applies not only to teaching but also to assessment [10]. Turning the corner on this notion towards a more inquisitive, learner-centric, and evidence-based framework has been challenging, but efforts towards competency based education are increasing [21]. Further opportunities and initiatives are many and include working together with local faculty members towards more holistic and competency driven frameworks for assessment. Programmatic integration of additional opportunities for assessment in simulated clinical scenarios is an ongoing program improvement initiative. Simulation in this context include high fidelity, low fidelity, and virtual scenarios, as well as inter-disciplinary opportunities focused on communication and quality improvement. Additional opportunities include working together to improve content and context for the Rosh question bank, focusing on clinical context such as tropical disease and appropriate diagnostic studies in the Emergency Department relevant to India and other global contexts.

The results of this study, while still on a small scale, indicate that a new generation of learners focused on learning and application to clinical practice may be emerging. The true outcome of change in clinical practice or clinical metrics has not been shown, but a changing framework of teaching and learning is one step in the right direction.

### Limitations

While conducted across program sites and various regions of the country, this study was conducted within an existing program framework. The potential for biased participation of trainees based on their existing enrollment is possible, although the variation in participation across the different trainees suggests a variable and authentic uptake. The choice of an online quiz tool for formative assessment could be mistaken as only relevant for exam preparation, and exploration of other formative assessment tools in this context could provide more information. Sample size was not sufficient to reveal significant differences within the qualitative interviews among the different user groups. However, representation of different types of learners within the interview groups did provide diversity in the responses. Interviews were conducted by external medical students, selected in part because of their prior lack of involvement with the program. More inclusion of local researchers in the study may have added local knowledge and context, although this may have also imparted bias related to program roles. The application of this study in only one country is unique and may limit replicability in other cultural contexts.

## Conclusions

The introduction of a FA tool into existing EM education and training programs in India has been successful. Trainees have seen improved exam scores when using the tool and report important perspectives on clinical knowledge uptake and application to clinical practice as reasons for utilization of the tool. Next steps will include strategies to broaden uptake even among our own learning population, given the significant variability in learner groups. Further study either within India or in other countries provides an additional opportunity to broaden understanding and potential impact.

### Electronic supplementary material

Below is the link to the electronic supplementary material.

Supplementary Material 1

## Data Availability

Not relevant.

## Declarations

### Authors’ information

N/A.

## Abbreviations

EM: Emergency Medicine
FA: Formative Assessment
