# Supplementary material for: Introduction of a formative assessment tool in a post-graduate training program in India: a mixed methods evaluation
Source: Int J Emerg Med. 2024 Mar 1;17:32. doi: 10.1186/s12245-024-00604-6 (PMC10908017; doi:10.1186/s12245-024-00604-6)
Supplement: Supplementary file 1 — Supplementary Material 1 [file 12245_2024_604_MOESM1_ESM.pdf]

Semi-Structured Interview Guide

Demographics:

Site
Gender
Age
Resident (PGY year) or Faculty member

The goal of this conversation is to learn more about your own study practices, as well as the introduction of Rosh Review as a new learning tool into the program. This year was the first 'formal' year of using Rosh Review, and while we expect that you 'liked' it, we want to learn more about your experience, how you used the tool, what you think could have been different or better.

As a starting point, can we start by gaining a better understanding of your learning patterns.

1. What motivates you to study?
    - Learning information for patient care, passing the test or exam, being successful in a program?

2. How do you best learn?
    - From books? From websites? From blogs? From exams? From patients

3. How do you use Rosh Review? How are you finding the experience of using Rosh Review? What do you use it for (assessment tool vs. learning tool)?
    - If yes – follow the conversation
    - If no – can you share why you don't use it? What do you use instead?

4. What do you like about using Rosh Review? How does it help you? Can you give me an example of a time that you have found it helpful? Can you relate it to your clinical practice?

5. What do you not like? Is there anything about it that makes you uncomfortable? Give me an example of a time that was uncomfortable? (*can talk about connectivity issues but try to find other issues as well*)

6. Have you used outside resources while working on your quizzes?

7. Do you study in a group when using Rosh Review?

8. How would you define success when using Rosh Review? Is it percentage, or learning or number of questions, or success in/application to clinical practice?

9. How would you compare your experience with Rosh review in this past year to prior years with the MEM program and/or other academic programs you have participated in? What did you like? What did you find challenging?

10. Any other thoughts/questions for me?
